# Risk of Viral Infectious Diseases from Live Bats, Primates, Rodents and Carnivores for Sale in Indonesian Wildlife Markets

**DOI:** 10.3390/v14122756

**Published:** 2022-12-10

**Authors:** Thais Q. Morcatty, Paula E. R. Pereyra, Ahmad Ardiansyah, Muhammad Ali Imron, Katherine Hedger, Marco Campera, K. Anne-Isola Nekaris, Vincent Nijman

**Affiliations:** 1 Oxford Wildlife Trade Research Group, School of Social Sciences, Oxford Brookes University, Oxford OX3 0BP, UK; 2 Department of Ecology, Institute of Biosciences, Federal University of Rio Grande do Sul, 9500 Ave., Bento Gonçalves 90010-150, Brazil; 3 Little Fireface Project, Cipaganti 44163, Indonesia; 4 Forest and Nature Conservation Policy Group, Wageningen University, 6708 PD Wageningen, The Netherlands; 5 Faculty of Forestry, Universitas Gajah Madah, Yogyakarta 55281, Indonesia; 6 Faculty of Life Sciences, Oxford Brookes University, Oxford OX3 0FL, UK

**Keywords:** zoonosis, Nipah, One Health, pandemic, COVID-19, wildlife trade, wet market, mammals

## Abstract

Southeast Asia is considered a global hotspot of emerging zoonotic diseases. There, wildlife is commonly traded under poor sanitary conditions in open markets; these markets have been considered ‘the perfect storm’ for zoonotic disease transmission. We assessed the potential of wildlife trade in spreading viral diseases by quantifying the number of wild animals of four mammalian orders (Rodentia, Chiroptera, Carnivora and Primates) on sale in 14 Indonesian wildlife markets and identifying zoonotic viruses potentially hosted by these animals. We constructed a network analysis to visualize the animals that are traded alongside each other that may carry similar viruses. We recorded 6725 wild animals of at least 15 species on sale. Cities and markets with larger human population and number of stalls, respectively, offered more individuals for sale. Eight out of 15 animal taxa recorded are hosts of 17 zoonotic virus species, nine of which can infect more than one species as a host. The network analysis showed that long-tailed macaque has the greatest potential for spreading viral diseases, since it is simultaneously the most traded species, sold in 13/14 markets, and a potential host for nine viruses. It is traded alongside pig-tailed macaques in three markets, with which it shares six viruses in common (Cowpox, Dengue, Hepatitis E, Herpes B, Simian foamy, and Simian retrovirus type D). Short-nosed fruit bats and large flying foxes are potential hosts of Nipah virus and are also sold in large quantities in 10/14 markets. This study highlights the need for better surveillance and sanitary conditions to avoid the negative health impacts of unregulated wildlife markets.

## 1. Introduction

Infectious diseases are responsible for more than 7 million deaths annually, causing negative impacts on global health and substantial economic losses [1,2]. Seventy-five percent of all emerging infectious diseases are zoonotic, i.e., diseases that have originated from an animal and crossed the species barrier to infect humans, of which many have their origins in wildlife [3,4]. Most of those emerging zoonotic diseases are caused by viruses. All recent pandemic diseases allegedly originated from wildlife, such as HIV, SARS, and COVID-19, are caused by viruses with a long history of adaptation to their natural hosts, suggesting that investigations about activities that bring wild animals and humans in close contact are urgently needed [5]. 

Global trade and commerce, including wildlife trade, are recognized as key factors to the increase in emerging viral infectious diseases [6,7,8]. Specifically for wildlife, trade usually involves close contact between humans and animals (or their products) during the harvest, processing and exchange, raising the risk of a zoonotic pathogen crossing species lines [9,10]. The potential for viral infections in wildlife markets is enhanced because animals are slaughtered on the spot to be either legally or illegally traded as medicines, meat and pets [11]. These wild animals are often originated from areas many hundreds of miles far from the market [12,13], are offered for sale alongside domestic animals and kept in cramped conditions with little regard for hygiene or welfare. In addition, during transportation or sale, species that would not naturally have contact with each other are often kept close together in the facilities [14,15]. Those contacts break existent geographical, ecological or behavioural separations of humans and domestic animals with wildlife, increasing the likelihood of cross-species pathogen transmission [16]. 

In recent decades, increased global human population and recently established domestic and international travel networks have escalated the commercialization of wildlife, creating a situation in which the extent and velocity of zoonotic pathogen movement are historically unmatched [17,18]. Much of the wild species are traded illegally, but even for most of the legally sold there is no mandatory testing for pathogens [19]; this means that once a pathogen has crossed the species boundary, the risk of the infection spreading to susceptible populations is elevated [20]. 

For instance, the outbreak of SARS-CoV, linked to civets in China’s wildlife markets, spread to 37 countries, affected 8,096 people (774 died) [21], and is estimated to have led to the loss of $40 billion to the global economy [22]. Moreover, in China, natural infections by SARS-CoV were detected in wild-caught masked palm civets (*Paguma larvata*) for sale in wildlife markets but were not present in farmed civets [23]. Examples of zoonotic diseases related to wildlife markets from the past few decades also include the Ebola virus in primates, monkeypox in African rodents and possibly HIV in chimpanzees (*Pan troglodytes*) [24,25]. 

China and South and Southeast Asia are considered global hotspots of emerging zoonotic diseases [26]. In those regions, wildlife is commonly traded in open markets; these markets have been considered ‘the perfect storm’ for zoonotic disease transmission. Events of pathogen transfer to humans could be avoided or greatly reduced if transmission was better understood and practices adjusted to mitigate risk, but the composition of species sold in markets and the potential cross-transmission of pathogens among them and to humans is still poorly investigated. In this study, we surveyed 14 wildlife markets in 10 cities of Indonesia to estimate volumes and composition of live wild species on sale and assess the potential of wildlife trade in disseminating zoonotic diseases and facilitating a spill over of viruses to humans and across wild species. 

## 2. Materials and Methods

We collected data on wildlife trade in markets located on the islands of Java and Bali, Indonesia. There are at least 53 animal markets on those islands, viz. nine large (50 to over 200 stalls or shops), 22 medium (20–49 stalls or shops) and 22 small (less than 20 stall or shops) [27]. For this study, we surveyed 14 markets of 10 cities ranging from 15 to 100 stalls that were visited for a total of 179 times, an average of 14.1 ± SE 3.5 times each over the period of February 2016–February 2020 (Table 1). Here, we focused on four mammalian Orders (primates, bats, rodents and carnivores) due to their phylogenetic relatedness with humans, high prevalence in the markets, and for being among the main mammalian orders hosting viruses, meaning that the susceptibility of a pathogen cross-transmission among them and with humans is more likely [28]. Each market was visited by one or two of the authors. By slowly walking through the market all live animals on sale (excluding domesticated ones) were identified and counted. Species identification was normally done in situ, mostly at the species level or, less frequently, at the genus level. This information was logged into a mobile phone in the market or recorded in a notebook after leaving the market. We also counted the number of stalls selling wild mammals as a measure of the size of the market. Trade was open and there was no need to resort to undercover techniques; no animals were purchased. 

Information on viruses potentially infecting the different species on sale was obtained from Johnson et al. [16], who catalogued the presence (or lack thereof) of 139 zoonotic viruses in 5,335 wild terrestrial animal species. In cases when the animals were identified at the genus level, we considered those species with known distribution in Java and Bali and summed up the number of viruses species for the genus. 

We summed the number of taxa on sale as a measure of species richness, and for each taxon we calculated the mean number and standard deviation of animals detected across all markets where they were present. We used Generalized Linear Models (GLM) with Gamma family of distribution to assess the relationship between the population size (in ln scale) of the city surveyed and both mean number of animals and richness of taxa on sale in the markets, using individual markets in each city as replicates. Similarly, we used GLMs to assess the relationship between the number of stalls selling wildlife in each market (in ln scale) and both the mean number of individuals and richness of taxa on sale. GLMs were performed using the “gamlss” package. We obtained the human population size in 2020 of each surveyed city from Statistics Indonesia [29]. To build the interaction network representing the interactions between markets and animals and the main associated diseases, a weighted matrix was constructed with the number of individuals that was recorded in each market. In order to visualize the animals that are traded in the markets and the potential diseases that animals carry and share, we built a diagram using the Sankey Network function of the “networkD3” package [30], where the animal taxa sold, market and virus species are the nodes, and the links are the number of animals offered for sale. All statistical analyses were performed in R statistical software v4.1.0 (R Foundation for Statistical Computing, Vienna, Austria) [31].

## 3. Results

### 3.1. Animals Sold in Markets

**Figure 1:**
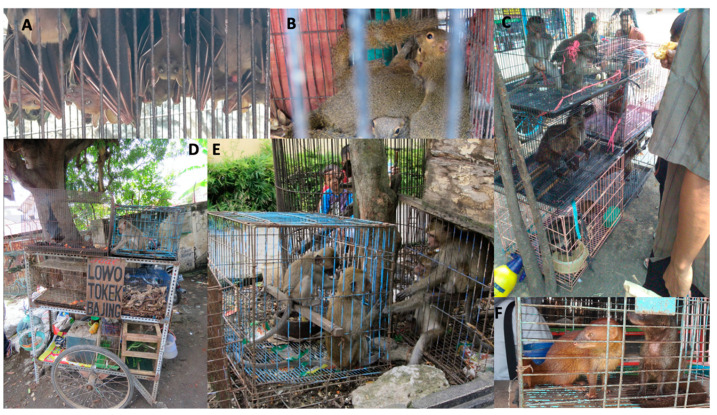
Trade in wild mammals in Java, Indonesia. (**A**) Indonesian short-nosed fruit bat (*Cynopterus titthaecheilus*); (**B**) plantain squirrel (*Callosciurus notatus*), (**C**) long-tailed macaque (*Macaca fascicularis*) and Asian palm civet (*Paradoxurus hermaphroditus*); (**D**) long-tailed macaque and giant fruit bat (*Pteropus vampyrus*); (**E**) long-tailed macaque; (**F**) Javan mongoose (*Urva javanica*).

We recorded 6,725 wild animals of at least 15 species within the order Rodentia, Chiroptera, Carnivora and Primates for sale in the 14 wildlife markets (Figure 1; Table 2). Five taxa accounted for almost 90% of traded animals, namely long-tailed macaque (*Macaca fascicularis*) (24%), Asian palm civet (*Paradoxurus hermaphroditus*) (22%), and plantain squirrel (*Callosciurus notatus*) (20%), followed by the large flying fox (*Pteropus vampytus*) (13%) and Indonesian short-nosed fruit bat [*Cynopterus titthaecheilus*; possibly in western Java also greater short-nosed fruit bat (*Cynopterus sphinx*) (10%)]. Informal conversations with sellers indicate that most of the animals must have been collected within Indonesia, mostly on Java, Bali and Sumatra, but also Borneo and possibly Sulawesi. None, or very few may have come from abroad.

**Figure 2:**
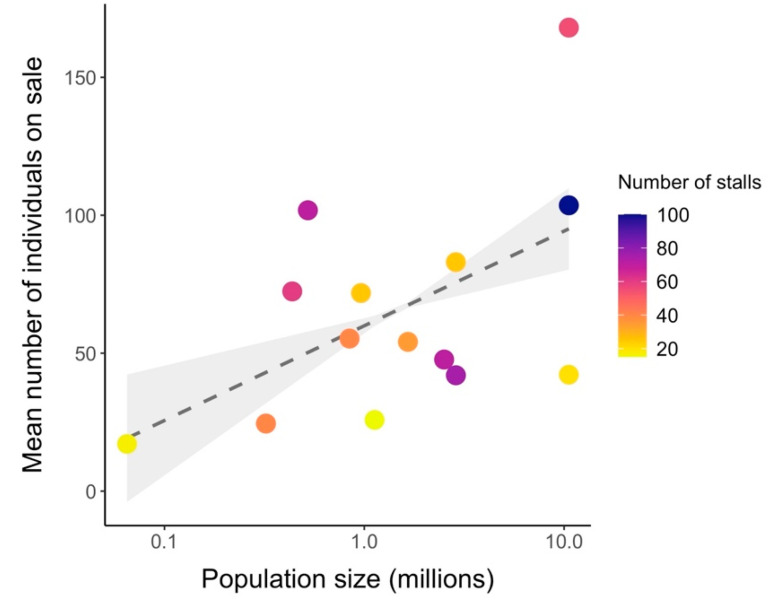
Relationship between human population size (in ln scale) of the surveyed city and mean number of primates, rodents, bats and carnivores on sale in wildlife markets in Indonesia. Each point is a sampled market, and the colour gradient refers to the estimated number of stalls selling wildlife in each market.

The mean number of individuals offered for sale showed a positive trend related with the human population in the respective city (Estimate = 14.91 ± 4.10, t-value = 3.64, *p*-value = 0.004) and the number of market stalls selling wildlife (Estimate = 39.54 ± 14.02, t-value = 2.82, *p*-value = 0.02) (Figure 2). Conversely, no clear trend was found between human population (Estimate = 0.53 ± 0.44, t-value = 1.21, *p*-value = 0.25) or number of stalls in the market (Estimate = 0.05 ± 1.45, t-value = 0.035, *p*-value = 0.97) with taxa richness on sale.

### 3.2. Virus Species and Network of Markets and Hosts

At least eight (53%) out of 15 animal taxa recorded for sale are hosts of virus species listed in the database consulted for known zoonotic diseases (Table 2). We recorded 17 different zoonotic viruses that can affect these taxa. Of those, nine viruses can infect more than one recorded species as a host, including Influenza A, Nipah, Cowpox, Herpes B; these viruses are all easily transmitted to humans with no need of vectors. 

The taxa with the highest number of zoonotic viruses recorded are both macaques; the long-tailed macaque, a potential host of 9 viruses (53% of the total number recorded), including Cowpox, Dengue, Hepatitis E, Herpes B, Monkeypox, Reston, Ebola, Simian Foamy, Simian retrovirus type D and Vesicular stomatitis viruses; and southern pig-tailed macaque, with 6 (35%) virus species, including Cowpox, Dengue, Herpes B, Simian Foamy, Simian retrovirus type D and St. Louis encephalitis viruses. Short-nosed fruit bats also stand out by being potentially infected by 5 (29%) of the viruses recorded, but different species from those infecting macaques, which include Influenza A, Issyk-Kul, Japanese encephalitis, Kyasanur forest disease, and Nipah viruses. Of the remaining mammal taxa sold, masked palm civet is a host of SARS-CoV (and SARS-CoV related) virus, Javan leopard cat is a host of Influenza A virus, Javan mongoose is a host of Hepatitis E virus and Rabies, large flying fox is a host of Nipah virus, and *Trachypithecus* langurs are a host of Dengue virus. No virus was reported for the two recorded squirrels (Prevost’s squirrel and plantain squirrel) and two civets (masked palm civet and small Indian civet) on trade, neither for Asiatic small-clawed otter (*Aonyx cinereus*), Javan ferret badger (*Melogale orientalis*) and slow lorises.

**Figure 3:**
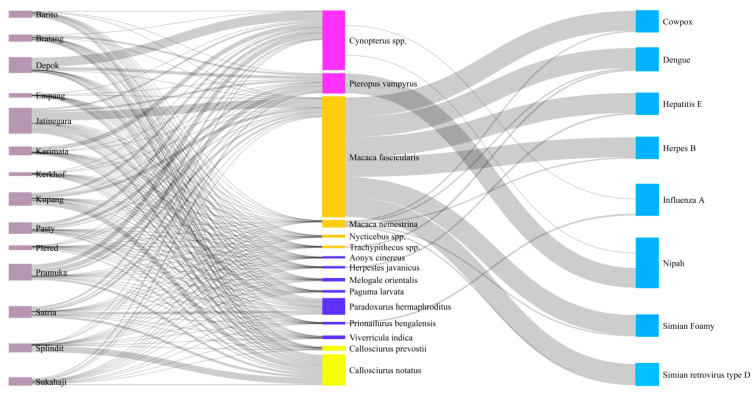
Sankey network diagram illustrating the 14 surveyed wildlife markets (purple), the mammal taxa (pink = bats, orange = primates, navy blue = carnivores, yellow = rodents), and the viruses (blue) these animals have the potential to host and share by co-occurring in the same markets.

The Sankey network diagram shows that long-tailed macaque is the species with the greatest potential for spreading diseases (Figure 3), since it is both a host for a large number of viruses and the most traded species, being sold in large quantities in 13 (93%) out of 14 surveyed markets.

In addition, long-tailed macaques are traded simultaneously with pig-tailed macaques in three markets, with the potential to spread six diseases in common (Cowpox, Dengue, Hepatitis E, Herpes B, Simian foamy, and Simian retrovirus type D). Indonesian short-nosed fruit bats and the large flying fox are also sold in large quantities and traded together in 10 markets, increasing the potential for the spread of Nipah virus. Furthermore, short-nosed fruit bats and the Javan leopard cat are traded in four markets in common and are potential hosts of Influenza A.

## 4. Discussion

### 4.1. Links between Wildlife Trade and Transmission of Viruses

Using the widespread and open trade in live wild mammals in markets on the two main islands of Indonesia as our case study, we show that live wildlife markets may provide optimal conditions for the spill over and spread of viral diseases. More populated cities and larger markets had larger quantities of animals being sold, and those same markets had on sale several mammal species that potentially share viruses in common. In addition, some of the most recorded species, such as long-tailed macaque and short-nosed fruit bats, are hosts of several virus species and are traded in large quantities across almost all markets, posing a risk of disease outbreaks to millions of people inhabiting those highly populated islands. These findings are of great concern in terms of public health, because there is a higher likelihood for an infected animal to end up in the market of a city where (i) there is a wider range of species, increasing the chance of a spill over, and (ii) that is more populated by humans, increasing the chance of fast pathogen spread and consequently for outbreaks and epidemics to occur. 

The number of zoonotic viruses potentially hosted by the traded species (17) in this study is very similar to the number (16) identified in wildlife traded as wild meat in Malaysia [32]; of these, nine are common between studies. This means that the risk of these viruses in infecting humans by contact in wildlife markets is not exclusive of Indonesia, but potentially widespread across Southeast Asia. For most zoonotic viruses reported here, the type of contact that sellers, buyers and even visitors have with the animals is enough to enable transmission. SARS-CoV, Influenza, Hepatitis E, Issyk-Kul, Nipah viruses can be transmitted through infected respiratory secretions and/or exposition to contaminated faeces and urine [33,34], which is very likely to occur in a crowded market, often with low levels of hygiene [35]. Others, such as Rabies, Cowpox, Herpes B Cercopithecine herpesvirus 1, Simian Foamy and Simian retrovirus type D can be transmitted transcutaneous through animal bites and scratches, which are also possible to happen when handling the animals or by humans having their mucous membranes or damaged skin exposed to animal body fluids [36]. Only 4/17 (23.5%) of the viruses identified cause vector-borne diseases, requiring the presence of a vector to be transmitted from infected animals to humans. These include Dengue, Japanese encephalitis and St. Louis encephalitis, which have mosquitoes as a vector, and Kyasanur forest disease virus that needs ticks as vectors to be transmitted [37,38]. Although it is not impossible for vectors to be present at the markets or, later, at the place where the bought animal is kept (especially ticks carried by the animals), it is reasonable to assume that the need of a vector decreases substantially the chance of those diseases to occur due to wildlife trade.

It is of great concern that the recorded virus species are potentially circulating in wildlife markets in Southeast Asia, since among them are some viruses responsible for causing serious and deadly diseases to humans. Nipah virus, in particular, is a bat-borne virus that has caused severe disease outbreaks in Asia, with mortality rates reaching over 90% in some cases, consisting of one of the deadliest viruses affecting humans [39]. This virus causes acute respiratory infection and fatal encephalitis, and yet there is no treatment for infected individuals or vaccine available [40]. The virus can spill over to domestic animals, such as pigs, horses, cats and dogs. In the surveyed markets in Indonesia, domestic animals, mostly dogs and cats, were seen being sold as pets in large quantities; free roaming cats and dogs, in addition to rats, are frequently encountered in the markets. These animals may be infected especially by contact with traded bats’ urine. Outbreaks of Nipah have happened in Malaysia, Bangladesh and India due to contact of humans with infected domestic animals and contaminated food [40,41]. Therefore, Indonesia and other countries presenting fruit bats of the Pteropodidae family may be at a very high risk, especially with the facilitation of spill over through trade of these species. Herpes B Cercopithecine herpesvirus 1 is also a pathogen worth of concern, since apparently healthy macaques can host it without any overt signs of disease [36], increasing the chance of infected animals to end up in the market and to be sold. Stress or immunosuppression, both common health issues due to poor enclosure conditions, was observed to increase the chance of macaques in shedding the virus [42]. Conversely, Herpes B in humans usually results in fatal encephalomyelitis or severe neurologic impairment, with a death rate of >70% when there is limited availability of antiviral therapy [36,42].

### 4.2. Sanitary and Health Implications

A number of species we encountered in the markets are included on Indonesia’s list of protected species and no wild-caught individuals are allowed to enter the trade. These include the Javan leopard cat, slow lorises and *Trachypithecus* langurs. In Indonesia trade in species that are not legally protected is regulated through a harvest quota system [43]. For mammals these are mostly set at zero (i.e., no wild harvest is allowed) or only small numbers are allowed to be harvested and traded for specific purposes. The numbers we observed in the markets greatly exceeded these harvest quotas, as for instance for the year 2020 a total of one small Indian civet was allowed to be traded for pets for all of Indonesia, in addition to five Prevost squirrels, five large flying foxes, six masked palm civets, 29 palm civets and 135 plantain squirrels. Single visits to the animal markets on Java and Bali often recorded these species in numbers far exceeding this. Hence, most of the trade in wild mammals in the markets in Java and Bali is illegal and in violation of Indonesia’s domestic legislation and regulations. The maintenance of an illegal trade not only increases the number of species and individuals on sale, creating more situations of contact for potential sharing and spreading of pathogens, but also hamper proper control and establishment of sanitary and hygienic measures to avoid viral transmissions and infections. 

While the illegal trade certainly aggravates the potential of wildlife markets in spreading diseases, the legal trade, if not properly monitored, also poses a similar threat to humans. Reducing this threat is not a simple task, once ownership, consumption and trade of wild species are usually part of the local culture, play important roles in local economy and occur more frequently in developing, but megadiverse, countries where surveillance is often insufficient [44,45]. It is, therefore, essential to consider this issue in the light of One Health approach and reinforce the appreciation that human, animal and ecosystem health are interdependent [46]. The implementation of measures to prevent new viral emergences and protect human health requires a holistic approach that embraces all three components. Wild population declines due to overexploitation and reduction in wildlife habitat quality were strongly related to a higher risk of disease transmission of animal viruses to humans [16]. Accordingly, any proposals should to integrate these multiple dimensions and take into account environmental, social and economic issues.

Sanitary measures should focus on the most affected species and largest markets and cities. For instance, the most commercialized species in our study, the long-tailed macaque, a potential host of nine virus species, has been recently classified by IUCN as Endangered [47], especially due to high utilization by humans as a meat source for local populations. During the COVID-19 pandemic, the demand for the species increased [47,48], consequently raising the risk of disease transmission. In addition, the species is being threatened due to its widespread use for biomedical and toxicological research [48]. Hence, most efforts should be done for controlling its trade and prevent viral diseases to spread from its use. However, for a more comprehensive analysis of risks, more information should be available on the pathogen loads in traded animals, transmission risks at different contact points, and potentials for animals to be taken to different parts of the country. One suggestion is an implementation of zoonotic vigilance, where species in markets can be periodically monitored for early detection of occurrence of main pathogens, allowing the prevention of outbreaks in both humans and animals [49,50]. A functional early alert system may attenuate the health, social and economic impacts of epidemics and pandemics [51].

In 2021, the World Health Organisation (WHO), the World Organisation for Animal Health (OIE) and the United Nations Environment Programme (UNEP) have issued guidance to help reduce public health risks associated with the sale of live wild mammals [52], prompted by the COVID-19 pandemic and the allegedly role of a wildlife market in Wuhan, China, in its origin. Although this attribution has been questioned, between 2017 and 2019 around 47 thousand individuals from 38 species were kept under poor welfare and hygiene conditions and sold in Wuhan’s markets prior to the COVID-19 pandemic [53]. This fact shows the potential of these markets in breaking the barriers of contact between humans and wild animals, even in large urban areas of the world. The guidance from WHO is focused on the sale of live species in food markets, but some may apply to the sale of live species for broader purposes, such as pet ownership. Among the measures are the obvious improvement of standards of hygiene and sanitation in these markets, which may include avoidance of keeping animals in overpopulated cages and regular cleaning and disinfection of animal enclosures, pest control and waste management with special attention to animal urine, faeces and other secretions. Traceability of farmed wild animals, where this is an option, can also contribute to curb the trade of animals illegally sourced from the wild that are more likely to be shedding a pathogen [52]. The document also recommends the development and implementation of campaigns for market traders, stallholders, consumers and the wide general public that can bring information about the risks of transmission of zoonotic pathogens at the human–animal interface, safety practices in handling and keeping live wild animals and what to consider when selling or buying an animal in order to reduce the likelihood of spreading zoonotic diseases.

In addition to wildlife hunting and trade, the modification of environments by deforestation, intensification of agricultural production and urbanization increases the possibility of interspecies transmission due to higher probability of contact between humans and animals [54,55]. In fact, forestation has been advocated as one potential ecological measure to prevent virus outbreaks [56]. Climate change may also expand the habitat range of some of the recorded taxa, such as bats, resulting in modifications in interactions among species and facilitating cross-species spillover [57]. Our study demonstrated the potential of applying interaction networks to better visualize the wildlife trade in Indonesia, as well as the potential diseases that may emerge through interactions between organisms. In the face of manyfold environmental changes happening in the world, new studies could use this approach to advance the understanding of the use and trade of animals in other regions of Asia, as well as other tropical areas in Africa and Latin America that are considered infectious disease hotspots [3,58].

In this study we highlight the need for better surveillance and sanitary conditions to avoid the negative health impacts of unregulated wildlife markets. More than half of the species traded in wildlife markets in Indonesia are in fact hosts of zoonotic virus species. This study could be used in the development of public health strategies in Southeast Asia, such as implementing sanitary measures and standards in wildlife markets. This information is also useful to develop awareness campaigns to educate people about the numerous health risks from trading or buying wildlife highlighted, encouraging them to buy wildlife that are legally sourced and surveilled for pathogens, or the consumption of alternative foods whenever possible. Such initiatives could have additional benefits for the conservation of threatened species by helping reduce the illegal domestic and international trade of species in and from Southeast Asia [59].

## Figures and Tables

**Table 1:** Details on location (city) with population size, names of the markets sampled, the sample size in number of visits to the market, number of stalls, and richness of mammalian taxa sold in each of the 14 wildlife markets surveyed in Indonesia (Java and Bali). Cities are listed from west to east.

City	Population Size (Million)	Market (Number of Stalls)	Visits (N)	Richness of Taxa (N)
Jakarta	10.562	Pramuka (100)	16	10
		Jatinegara (55)	28	15
		Barito (20)	28	14
Bogor	1.127	Tj Empang (15)	5	6
Bandung	2.510	Sukahaji (40)	40	13
Garut	0.065	Kerkhof (17)	35	11
Cirebon	0.322	Plered (40)	8	10
Semarang	1.654	Karimata (35)	10	9
Yogyakarta	0.436	Pasty (60)	7	10
Surakarta	0.522	Depok (70)	7	4
Surabaya	2.874	Bratang (75)	2	7
		Kupang (25)	2	8
Denpasar	0.963	Satria (25)	6	8
TOTAL		(647)	179	15

**Table 2:** Average number of individuals sold in the 14 wildlife markets surveyed in Indonesia, and number of zoonotic viruses that are able to infect these taxa as hosts.

Taxon	Number of Individuals (Mean When Present ± SD)	Number of Markets with Presence (% of Total)	Number of Zoonotic Viruses (% of Total)
Plantain squirrel *Callosciurus notatus*	1313 (14.4 ± 11.0)	14 (100)	0 (0)
Prevost’s squirrel *Callosciurus prevostii*	115 (4.5 ± 1.6)	6 (43)	0 (0)
Masked palm civet *Paguma larvata*	46 (1.5 ± 0.6)	7 (50)	1 (6)
Javan leopard cat *Prionailurus bengalensis*	111 (1.8 ± 0.5)	6 (43)	1 (6)
Small Indian civet *Viverricula indica*	63 (1.7 ± 1.4)	11 (79)	0 (0)
Javan mongoose *Herpestes javanicus*	57 (1.2 ± 0.4)	9 (64)	2 (12)
Asian palm civet *Paradoxurus hermaphroditus*	1501 (7.5 ± 7.9)	14 (100)	0 (0)
Asiatic small-clawed otter *Aonyx cinereus*	45 (1.5 ± 0.8)	7 (50)	0 (0)
Javan ferret badger *Melogale orientalis*	40 (1.8 ± 0.7)	9 (64)	0 (0)
Indonesian short-nosed fruit bat *Cynopterus titthaecheilus*	656 (19.9 ± 17.9)	10 (71)	5 (29)
Large flying fox *Pteropus vampyrus*	907 (9.5 ± 9.0)	13 (93)	1 (6)
Long-tailed macaque *Macaca fascicularis*	1620 (10.3 ± 15.6)	13 (93)	9 (53)
Southern pig-tailed macaque *Macaca nemestrina*	70 (2.8 ± 1.9)	3 (21)	6 (35)
Slow loris *Nycticebus* spp.	141 (2.9 ± 2.8)	5 (36)	0 (0)
Langur *Trachypithecus* spp.	40 (2.2 ± 1.3)	5 (36)	1 (6)
Total	6725	14 (100)	17 (100)

## Data Availability

The data presented in this study that are not yet included in the paper are available on request from the corresponding authors.
